# Small Bowel Neuroendocrine Tumors with Inguinal Metastases: A Diagnostic and Therapeutic Dilemma

**DOI:** 10.7759/cureus.692

**Published:** 2016-07-14

**Authors:** Faisal Inayat, Kevin P Daly, Farhad Askarian, Muhammad W Saif

**Affiliations:** 1 Department of Medicine, New York-Presbyterian Hospital, Weill Cornell Medical College; 2 Department of Radiology, Tufts Medical Center, Tufts University School of Medicine; 3 Department of Pathology, Tufts Medical Center, Tufts University School of Medicine; 4 Hematology/Oncology, Tufts Medical Center, Tufts University School of Medicine

**Keywords:** neuroendocrine tumors, small bowel, inguinal lymph node, octreotide scintigraphy, 68ga-dotatate pet/ct

## Abstract

Small bowel neuroendocrine tumors (NETs) are frequently characterized by a strong propensity to metastasize to the liver, mesentery, and peritoneum. However, only a few extra-abdominal metastatic sites have been reported in the published literature. The present paper implicates that primary small bowel NETs may unusually metastasize to the inguinal lymph nodes. Furthermore, we discuss the formidable diagnostic and therapeutic challenges associated with the metastatic NETs.

## Introduction

Small bowel neuroendocrine tumors (NETs) are the most common malignancies of the small intestine. They are mostly indolent tumors (survival measured in years), yet resistant to cytotoxic chemotherapy [1-2] and, if advanced (surgically unresectable), usually fatal. Small bowel NETs commonly metastasize to the liver and mesentery; however, it is unusual that it will recur as an inguinal lymph node. Therefore, small bowel metastatic NET presenting as an inguinal mass can be the source of extreme diagnostic confusion, both clinically and pathologically.

## Case presentation

A 66-year-old male was diagnosed with a Stage IIIB small bowel NET three years previously. He underwent exploratory laparotomy and small bowel segmental resection, along with mesenteric resection of the mass. The tumor measured 2.3 cm in its greatest dimension. It involved the full thickness of the bowel wall. It was present in the mesentery, having a perineural invasion, with foci suspicious for lymphovascular invasion. However, after the curative resection, the proximal and distal margins of the small bowel wall were clear and free of tumor. Since then, the patient has been followed with annual imaging by staggered computed tomography and octreotide scintigraphy every six months. The disease had been in a deep remission without specific therapy. His chromogranin A (CGA), urinary 5-hydroxyindoleacetic acid (5-HIAA), and neurokinin A (NKA) remained within normal limits throughout, and there was no clinical evidence of recurrence until his current presentation.

The patient presented to our institution with night sweats and a weight loss of 20 lbs over the last three months. He denied fever, abdominal pain, diarrhea, flushing, constipation, and fatigue. His physical examination was unremarkable. On admission, laboratory studies revealed 5-HIAA 6.1 mg/24 hr (< 8) and CGA 36 ng/mL (< 93). Informed patient consent was obtained prior to treatment. No identifying patient information is contained in this paper.

**Figure 1:**
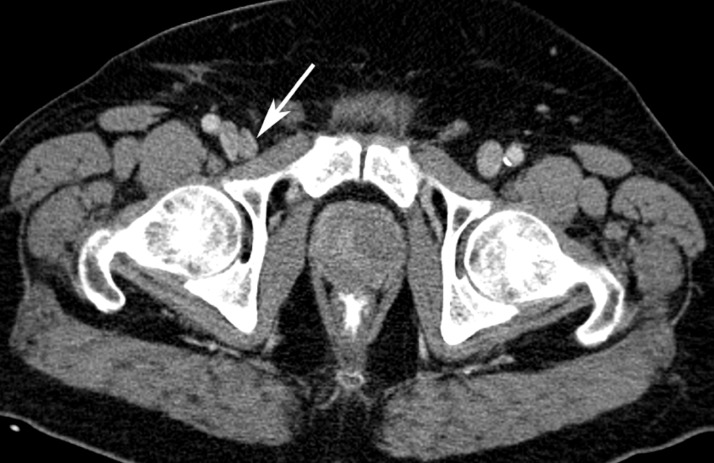
Computed Tomography (CT) Scan of the Pelvis An axial computed tomography image with intravenous contrast through the lower pelvis shows an enlarged right inguinal lymph node (white arrow) adjacent to the right common femoral vein.

Computed tomography (CT) through the lower pelvis showed an enlarged right inguinal lymph node adjacent to the right common femoral vein (Figure 1).

**Figure 2:**
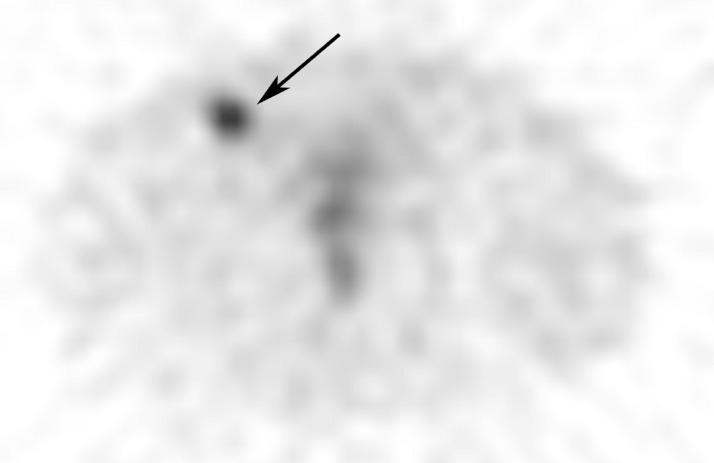
Octreotide Scintigraphy with Single-Photon Emission Computed Tomography (SPECT) An axial image obtained from single-photon emission computed tomography (SPECT) acquisition through the pelvis 24 hours following the IV injection of 5.9 mCi Indium-111 pentetreotide (OctreoScan, Mallinckrodt Pharmaceuticals, Maryland Heights, MO), a somatostatin analogue. An abnormal increased radiotracer uptake was identified (black arrow) in the right anterior lower pelvis corresponding to the enlarged right inguinal lymph node seen on the CT scan. This finding indicates nodal metastasis from the patient’s previously resected small bowel neuroendocrine tumor.

The patient subsequently underwent an octreotide scintigraphy. An axial image was obtained from a single-photon emission computed tomography (SPECT) acquisition through the pelvis 24 hours following the intravenous injection of 5.9 mCi Indium-111 pentetreotide (Octreoscan™, Mallinckrodt Pharmaceuticals, Maryland Heights, MO), a somatostatin analog. Abnormally increased radiotracer uptake was identified in the right anterior lower pelvis corresponding to the enlarged right inguinal lymph node seen on the CT scan (Figure 2). This finding indicated nodal metastasis from the patient’s previously resected small bowel NET.

**Figure 3:**
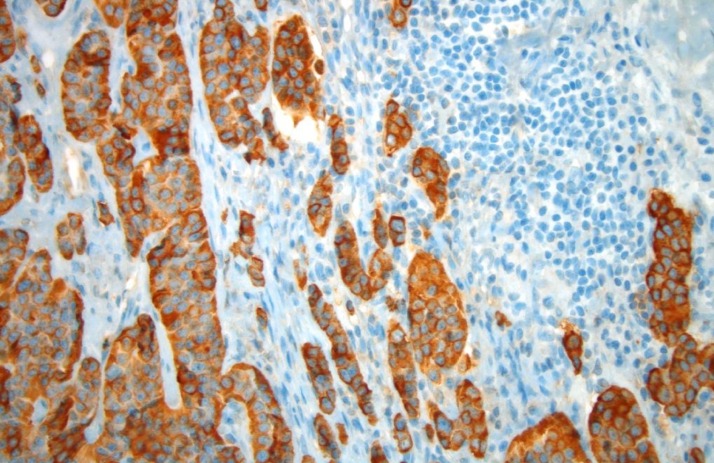
Image of Chromogranin A The picture of slide shows strong cytoplasmic staining for chromogranin A (neuroendocrine marker). This marker is more sensitive for low-grade than high-grade neuroendocrine tumors.

An uneventful biopsy of the right inguinal lymph node was performed. Histopathologic analysis of the specimen showed strong cytoplasmic staining for CGA (Figure 3).

**Figure 4:**
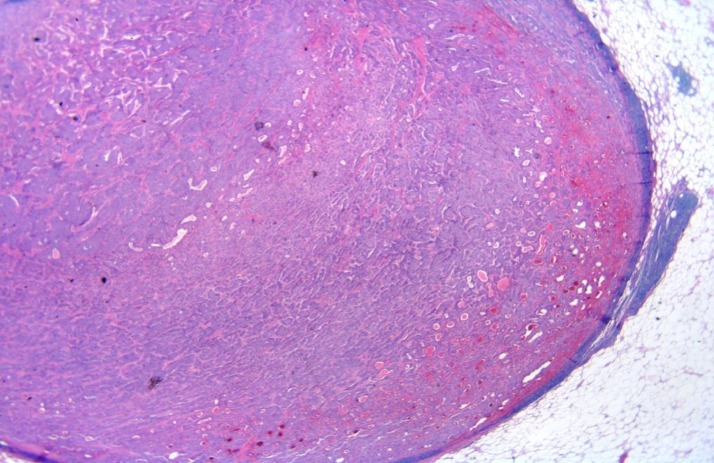
Image of H&E 2X The picture of slide shows a low power magnification of a lymph node, which was completely replaced by neuroendocrine tumor.

**Figure 5:**
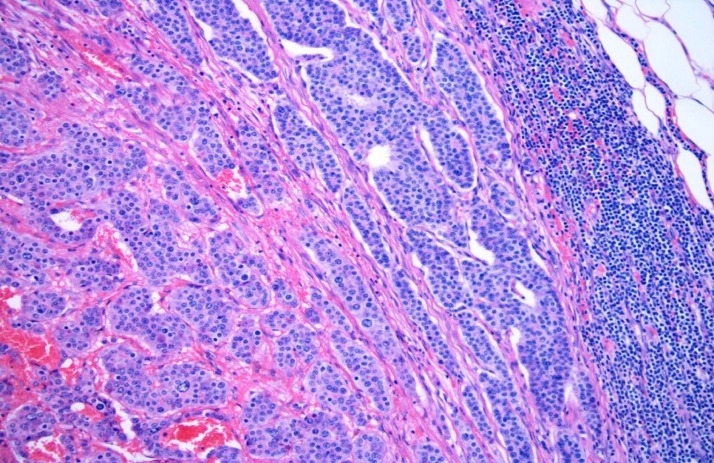
Image of H&E 40X The picture of this slide shows a high-power magnification of many clusters of neuroendocrine tumor. The lymph node was mostly replaced by the neuroendocrine tumor and had only a rim of lymphocytes.

The tumor had completely replaced the lymph node and there was only a rim of lymphocytes with many clusters of NET (Figures 4-5).

**Figure 6:**
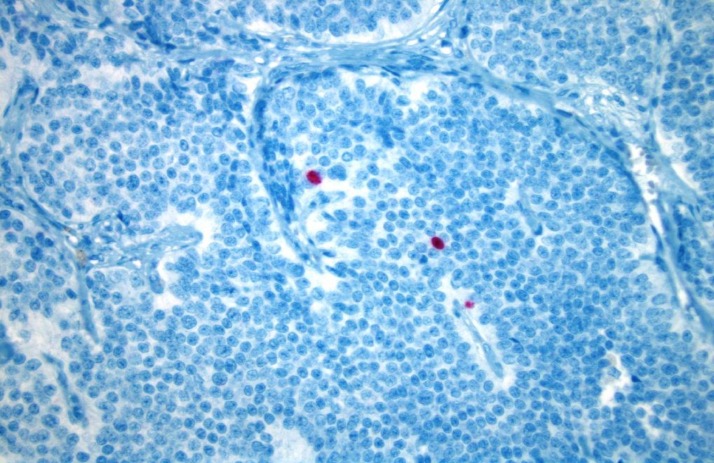
Image of Ki67 The picture of this slide shows rare nuclear staining for Ki67 (red color) consistent with a very low proliferation rate. This stain supports the low-grade nature of this neuroendocrine tumor.

The tumor showed nuclear staining for Ki67, which was consistent with a very low proliferation rate (Figure 6).

Histopathology confirmed the inguinal lymph node as metastatic NET from the small bowel.

The case was discussed in a multidisciplinary tumor board for this unusual right inguinal metastasis, and it was decided to closely monitor the tumor with octreotide scintigraphy every six months. At his one-year follow-up, there was no evidence of tumor progression.

## Discussion

Small bowel NETs are difficult to diagnose due to non-specific symptoms that are more often found in common gastrointestinal diseases. These neoplasms frequently involve the elderly patient population and are usually indolent, but early and specific diagnosis is important. 

NETs of the small bowel are generally located in the terminal ileum as a flat and fibrotic submucosal tumor commonly measuring 1 cm or less [1]. In one-third of the cases, multiple small lesions in the small bowel may arise owing to the adjacent lymphatic dissemination. Furthermore, these lesions have a strong predilection for metastasis to the liver [2]. A poor prognosis has been noted in patients having liver metastases. Other common metastatic sites associated with small bowel NETs include the mesentery and peritoneum. Mesenteric metastases are common and occur independently of the size of the primary tumor. These metastatic lesions are generally larger in size and are typically associated with marked mesenteric fibrosis, which may lead to partial or complete bowel obstruction. In addition to chronic obstruction, patients with peritoneal metastasis may experience weight loss and malnutrition [3].

In the literature, case reports are available highlighting the extra-abdominal metastases of small bowel NETs to the skeleton, ovaries, breasts, lungs, CNS, skin, and the peripheral lymph glands (supraclavicular lymph glands) [1, 3-4]. However, inguinal lymph node metastasis from primary small bowel NET is an unusual and extremely rare clinicopathologic entity. In our research, there has been only one case in the published literature regarding a carcinoid tumor metastasizing to the inguinal sac [5]. Hence, the present case appears to be only the second report of small bowel NET metastasis to right inguinal lymph nodes.

The correct identification of inguinal metastasis from small bowel NETs is difficult to ascertain, as these metastases are deemed clinically asymptomatic and diagnostic modalities do not necessarily demonstrate unique features distinguishing the lesion as primary small bowel malignancy. Therefore, the diagnosis is largely reliant on CGA, 5-HIAA, and circulating NKA, which are used as major laboratory diagnostic tests. However, our patient was unique in this regard as his CGA and 5-HIAA levels were within regular limits, and he was eventually diagnosed by octreotide scintigraphy and consistent histopathologic analysis of the lesion. Therefore, the results of biochemical markers for NETs may often be misleading or confusing [6]. 

The octreotide scintigraphy (111In-Pentetreotide) is a useful adjunct and likely the most commonly used diagnostic imaging modality for NETs. Recently, the 68Ga–tetraazacyclododecane tetraacetic acid–octreotate (68Ga-DOTATATE) PET/CT has been evaluated for the diagnosis and workup of NETs. It was equivalent or superior to 111In-Pentetreotide imaging in numerous studies and had no considerable adverse events [7-8]. Haug, et al. [7] investigated the effectiveness of 68Ga-DOTATATE PET/CT in the follow-up of patients after curative resection of NETs with promising results. Given the lack of significant toxicity, lower radiation exposure, and improved accuracy compared to 111In-Pentetreotide, 68Ga-DOTATATE imaging can be employed where available.

In terms of management of metastatic NETs, the PROMID study [9] showed that octreotide (Sandostatin LAR®), 30 mg intramuscularly, compared to placebo lengthened the time to tumor progression (14.3 versus 6 months) in patients with metastatic NETs. Additionally, radiation can be considered with a locally advanced neuroendocrine tumor [10]. In our patient, a multidisciplinary tumor board decided that the patient should be managed medically. In that context, close monitoring with octreotide scintigraphy was recommended every six months, as this detected the metastatic lymph node.

## Conclusions

Small bowel primary NETs can metastasize to inguinal lymph nodes, which may become a diagnostic and therapeutic predicament for clinicians treating this disease. Knowledge regarding such unusual metastatic sites of NETs is of paramount importance in order to diagnose cases early, monitor tumor progression, and to institute effective treatment. Furthermore, the present paper adds evidence to the previous studies that imaging modalities employed for monitoring purpose in cases with metastatic NETs may have higher sensitivity and specificity than biochemical markers.
